# Visualization of tumor heterogeneity by in situ padlock probe technology in colorectal cancer

**DOI:** 10.1007/s00418-017-1557-5

**Published:** 2017-03-20

**Authors:** Amin El-Heliebi, Karl Kashofer, Julia Fuchs, Stephan W. Jahn, Christian Viertler, Andrija Matak, Peter Sedlmayr, Gerald Hoefler

**Affiliations:** 1 Institute of Cell Biology, Histology and Embryology, Medical University of Graz, Harrachgasse 21/VII, 8010 Graz, Austria; 2 Institute of Pathology, Medical University of Graz, Graz, Austria

**Keywords:** In situ, Padlock probe, Tumor heterogeneity, NGS, Colorectal cancer

## Abstract

Tumor heterogeneity is considered a major cause for therapy resistance in colorectal cancer. Sub-populations of cells with different genetic alterations may exist in spatially distinct areas. Upon therapy, resistant sub-clones may enrich and ultimately lead to disease progression. Although ample data are available on tumors which are heterogeneous on a morphological level, only little is known about morphologically homogeneous tumors. We aimed to investigate if morphologically homogeneous colorectal cancer can harbor a heterogeneous genetic landscape. We chose to microdissect six morphologically homogeneous colorectal carcinomas into several areas and performed next-generation sequencing (NGS) to identify tumors with genetic heterogeneity. We then applied an mRNA-based in situ mutation detection technology based on padlock probes to localize and visualize mutations directly in the tumor tissue. In three out of six tumors, NGS revealed a high rate of variability of mutations between different tumor areas. We selected two cases for in situ mutation detection to visualize genetic heterogeneity. In situ mutation detection confirmed differences in mutant allele frequencies between different tumor areas of morphological homogeneous tumors. We conclude that genetic heterogeneity in morphologically homogeneous colorectal cancer is an observable, but underreported event. Our results illustrate the power of in situ mutation analysis to visualize genetic heterogeneity directly in tumor tissue.

**Electronic supplementary material:**

The online version of this article (doi:10.1007/s00418-017-1557-5) contains supplementary material, which is available to authorized users.

## Introduction

Colorectal cancer is a complex disease with greatly varying clinical responses to therapy (Ogino and Goel 2008; Blanco-Calvo et al. 2015). The different clinical responses are primarily explained by cancer cell hetereogeneity within a tumor mass (Misale et al. 2014). Tumor heterogeneity presents as a mixture of sub-populations of cancer cells with different genetic alterations which may exist in spatially distinct areas of a tumor (Marusyk et al. 2012; Bedard et al. 2013; El-Heliebi et al. 2014). These distinct tumor areas can harbor sub-populations of tumor cells which will be resistant to future therapy even before actual initiation of therapy (Diaz-Cano 2012; Heitzer et al. 2013). In the course of treatment, these cells can be enriched and ultimately lead to the emergence of clinical drug resistance (Misale et al. 2012). Therefore, it is of great clinical importance to identify genetically heterogeneous tumors. Parallel to genetic heterogeneity, colorectal cancer manifests in a variety of different morphological subtypes, such as glandular, mucinous, signet ring cell, and several others (Fleming et al. 2012). The different morphological phenotypes can be found as mixtures with each other in the same tumor, resulting in a morphological heterogeneous tumor (Fleming et al. 2012). Vice versa, tumors displaying primarily with only one morphological subtype can be regarded as morphologically homogeneous. Nevertheless, concerning their genetic landscape, homogeneous tumors could hide several sub-clones which might not be distinguished by the conventional light microscopy.

Although ample data on genetic heterogeneity have been described (Gerlinger et al. 2012; Nik-Zainal et al. 2012a, b; de Bruin et al. 2014; Zhang et al. 2014; Sottoriva et al. 2015), the investigated tumor tissues were not selected for their morphological homogeneous appearance. Data on the possible genetic heterogeneity of morphologically homogeneous tumors are currently lacking. In addition, these studies were based on next-generation sequencing (NGS), where tissues are lysed to extract nucleic acids and the histological context of the tissue is lost. There is a gap between sequencing data and spatial information. A solution to bridge the gap is the mRNA-based padlock probe technology (Larsson et al. 2010). Padlock probe technology can detect point-mutations in situ in the tissue section and retains the original location of the transcript. We hypothesize that morphologically homogeneous tumors may harbor genetic heterogeneity. Therefore, the aim of this study was to evaluate the genetic landscape of morphologically homogeneous colorectal tumors by combining NGS and a mRNA-based in situ approach. First, we manually microdissected morphologically homogeneous tumors into several distinct areas. Each area was analyzed using next-generation sequencing (NGS) to identify mutations with different mutation frequencies in separate tumor areas. Second, we visualized and quantified the detected mutations by a mRNA-based in situ approach directly in FFPE tumor tissue sections.

## Materials and methods

### Compliance with ethical standards

Approval of the institutional ethics committee was obtained for this study (Medical University Graz EK24-455 ex 11/12). All methods were performed in accordance with the relevant guidelines and regulations.

### Tumor samples

Computerized pathological reports from the Institute of Pathology, Medical University of Graz, Austria, were searched for surgical specimens of routinely reported colorectal cancers, resected at the Krankenhaus der Barmherzigen Brüder St. Veit/Glan, Austria, between January 2011 and January 2013. As the relevance of genetic tumor heterogeneity is primarily associated with the possible outgrowth of resistant sub-clones under tumor specific therapy, only tumors eligible in principle for adjuvant therapy (UICC stage III or IV) were included. All tumors were classified as invasive and all but one of these tumors displayed regional lymph node metastasis. Patients who had received neo-adjuvant treatment for rectal cancer were excluded from the study. Thus, 39 colorectal cancers were included for further evaluation. All tumors were formaldehyde (4% m/V buffered solution)-fixed and paraffin-embedded (FFPE) in their entirety. FFPE tissues were provided by the Biobank of the Medical University of Graz, Austria. Hematoxylin and eosin (HE) stained slides were evaluated for a homogeneous morphological appearance across the whole tumor area, without distinct foci delimitable on comparison with the surrounding tumor tissue by two pathologists (SJ, CV). Patterns of differentiation were based on the morphological patterns described for the special subtypes of colorectal carcinoma according to the “WHO Classification of Tumors of the Digestive System” such as mucinous, signet ring cell, micro papillary differentiation, etc (Bosman et al. 2010). The homogeneous tumors selected essentially demonstrated tubular, tubulopapillary, and occasionally cribriform architecture, which were present in similar proportions across the different tumor areas, without circumscript changes in differentiation patterns. After evaluation, six tumors with a homogeneous morphological appearance were selected (Supplementary Table 1). Five tumors were colon cancers and one tumor was a rectal cancer located in the proximal, peritonealized rectum. Serial sections with first and last section HE stained for topographical reference were obtained and tumor tissue was manually microdissected (scraped off) from unstained sections with a scalpel. Tumor tissue was dissected from 4 equally sized areas and DNA extracted (Maxwell, MDxResearch System-Promega, Fitchburg, Wisconsin, USA).

### Cell line

SW620 colon cancer cells were purchased from ATCC and were cultured in the media recommended by the distributor at 37 °C with 5% CO_2_. Cells were tested negative for mycoplasma and were verified to be SW620 by STR analysis using PowerPlex 16 System Kit (Promega, Mannheim, Germany).

### Ion torrent comprehensive cancer panel-sequencing of colorectal cancer tissue

Mutation analysis was performed using the Ion AmpliSeq Comprehensive Cancer Panel (Thermo Fisher Scientific, Waltham, MA, USA, Cat Nr 4477685) comprising 16,000 amplicons which cover the whole coding region of 409 tumor-related genes of the Wellcome Trust Cancer Gene consensus. Libraries were prepared using the Ion AmpliSeqTM Library Kit 2.0 (Thermo Fisher Scientific) using the recommended amount of 10 ng DNA in each PCR reaction and sequencing was carried out on an Ion Proton Sequencer (Thermo Fisher Scientific) (Rothberg et al. 2011). Emulsion PCR and sequencing runs were performed with the appropriate kits (Ion One Touch Template Kit v2 and Ion Proton 200 Sequencing Kit, Thermo Fisher Scientific) using Ion PI chips (Thermo Fisher Scientific). Sequencing length was set to 520 flows and yielded reads ranging from 70 to 150 base pairs consistent with the expected size range. On average, 20 million reads were obtained for each DNA library with consistently more than 80% of bases at AQ20. Data analysis was performed using the Ion Torrent Suite Software Plug-ins (Thermo Fisher Scientific, open source, GPL). Briefly, this included base calling, alignment to the reference genome (HG19) using the TMAP mapper and variant calling by a modified diBayes approach taking into account the flow space information. All called variants were annotated using open source software [Annovar (Wang et al. 2010), SnpEff (Cingolani et al. 2012)]. All coding and non-synonymous (amino acid changing) mutation calls present in tumor tissue and absent in the corresponding normal tissue were further evaluated and visually inspected in Integrative Genomics Viewer (IGV) (Robinson et al. 2011). Variant calls with an allele frequency greater than 5% in any of the samples were included in the analysis and further evaluated for heterogeneity in all samples. Variant calls resulting from technical read errors (e.g., homopolymers, inadequate primer removal) or sequence effects (e.g., short repeat sequences) were excluded from the analysis.

### Pyro-sequencing

For in situ mutation detection, the tumors K229, K255, and K386 were identified as the best candidates, as NGS data indicated genetic heterogeneity. Before applying in situ mutation detection, pyro-sequencing was performed of the tumors K229 and K386 to confirm NGS data. Pyro-sequencing was performed using the Qiagen Pyromark platform with the appropriate kits and according to the manufacturer’s recommendations. Pyro-sequencing assays were designed for the DPYD (R886H) (Tumor K229) and FANCD2 (E912Q) (Tumor K386) mutations using the Pyromark Assay Design software. Detection and quantitative mutation analysis were performed using the Qiagen Pyromark software.

### MSI analysis

Microsatellite stability was assessed using the Promega MSI Analysis system (MD1641, Promega, Madison, USA) which includes fluorescently labeled primers for co-amplification of seven markers for analysis of the MSI-high (MSI-H) phenotype, including five nearly monomorphic mononucleotide repeat markers (BAT-25, BAT-26, MON0-27, NR-21 and NR-24) and two highly polymorphic pentanucleotide repeat markers (Penta C and Penta D). Amplified fragments were detected using an ABI 3500 Genetic Analyzer.

### Sample preparation for padlock probe approach

FFPE sections (5 µm) were transferred to superfrost plus slides (Thermo Fisher Scientific) and baked for 3 h at 60 °C. Before applying the in situ mutation detection method, the tissue sections were deparaffinized and pre-treated in 2 mg/ml pepsin in 0.1 M HCl (Sigma) for 30 min at 37 °C. The digestion was stopped with DEPC-treated H_2_O (DEPC-H_2_O) for 5 min followed by a wash in DEPC-PBS for 2 min. Thereafter, the tissue was dehydrated using an ethanol series of 70, 85, and 99.5% for 1 min each and stored at −80 °C until use for in situ mutation detection. One tissue section of the colorectal cancer samples was HE stained for histopathological confirmation of the diagnosis and representation of different tumor areas. The HE stained slide was used for orientation prior to mutation scoring by in situ mutation detection. For the tumor K255, only areas 1 till 3 were available for in situ mutation detection, as there was no tumor tissue left in deeper sections of the tumor block.

For cell line experiments, SW620 were seeded with media onto superfrost plus slides (Thermo Fisher Scientific) over-night at 37 °C with 5% CO_2_. Cells were then washed with DEPC-PBS for 2 min, fixed in 4% m/V formaldehyde (Labonord, Templemars, France) for 15 min, washed with DEPC-PBS, and then dehydrated via a graded ethanol series and further processed as for tissue sections.

### LNA primer, padlock probe design, and detection probes

We designed an individualized patient-specific in situ assay based on the next-generation sequencing data.

The selected colorectal cancer cases were confirmed to carry a point mutation in the FANCD2 gene c.2734G > C (Tumor ID K386) (Substitution–coding) (Fanconi anemia complementation group D2) and the KAT6A gene c.1425A > C (Tumor ID K255) (Substitution–coding) (Lysine Acetyltransferase 6A).

Padlock probes were designed using the CLC Main Workbench software (CLC Bio Workbench Version 7.6, Qiagen; Venlo, Netherlands) according to the guidelines published by Weibrecht et al. (2013). A padlock probe for β-actin was used as a control (Grundberg et al. 2013). The mRNA sequence was retrieved from the National Center for Biotechnology Information [NCBI, GenBank accession number NM_033084.3 (FANCD2), NM_006766 (KAT6A)]. Depending on genotype, the designed mutation-specific padlock probes differ only in the last nucleotide at the 3′-end target sequences. All padlock probes were ordered 5′-phosphorylated (Integrated DNA Technologies; Coralville, IA, USA). LNA primers were purchased from Exiqon (Exiqon; Vedbaek, Denmark) and detection probes from Biomers (Biomers; Ulm, Germany). The LNA primer, padlock probe, and detection probe sequences are summarized in Table 1.

**Table 1:** Oligonucleotide sequences

Primers	Sequences (5′–3′)
ACTB*	C+GG+GC+GG+CG+GATCGGCAAAG
FANCD2_LNA	A+TG+GG+AA+TT+AT+GTAGTAACAATG
KAT6A_LNA	T+TT+CT+GC+AG+TG+CTTGTTCTT

Padlock probes	Sequences (5′–3′)
plp_ACTB*	AGCCTCGCCTTTGCCTTCCTTTTACGA**CCTCAATGCACATGTTTGGCTCC**TCTTCGCCCCGCGAGCACAG
plp_FANCD2_WT	AAAAGACATCATTGTTCCTAGTAAT**CAGTAGCCGTGACTATCGACT**GGTTCAAAGTCACAGGGAAGGAAG
plp_FANCD2_MUT	AAAAGACATCATTGTTTCCTTTTACGA**CCTCAATGCTGCTGCTGTACTAC**TCTTCTCACAGGGAAGGAAC
plp_KAT6A_WT	ATCATGACTGAGAAAGAAA**AGTAGCCGTGACTATCGACT**AATGCGTCTATTTAGTGGAGCCCACTATCTTTGGGAGCCAGGAA
plp_KAT6A_MUT	ATCATGACTGAGAAAGAAA**CCTCAATGCTGCTGCTGTACTAC**AATGCGTCTATTTAGTGGAGCCTTCTATCTTTGGGAGCCAGGAC

Detection probes	Sequences (5′–3′)
D1_ATTO 488	Atto 488-**CCTCAATGCACATGTTTGGCTCC**
D2_ATTO 647N	Atto 647N-**AGTAGCCGTGACTATCGACT**
D3_ATTO 550	Atto 550-**CCTCAATGCTGCTGCTGTACTAC**
D4_Cy3	Cy3-**AGTAGCCGTGACTATCGACT**
D5_FITC	FITC-**CCTCAATGCTGCTGCTGTACTAC**

Padlock probes were 5′-phosphorylated. The fluorophores of the detection oligos were 5′ conjugated

+: the following base is LNA modified; underlined: target complement sequence, bold: detection probe complement sequence

*Modified from (Grundberg et al. 2013)

The specificity and sensitivity of the–wild-type padlock probes were first tested in the cell line SW620 which is wild type for FANCD2 and KAT6A (data not shown). Since the mutations were patient-specific, mutation padlock probes could not be tested on cell lines. After confirmation of the quality of the probes, the in situ method with wild type and mutation padlock probes were applied on an FFPE-tissue section from colorectal cancer patients.

### In situ mutation detection in tissues and cell lines

All in situ reactions were performed in secure-seals hybridization chambers (Sigma). The reaction volumes for the tissue slides were either 50, 100, or 350 μl depending on sample size. The sample was rehydrated by incubating the wells with PBS-T [DEPC-PBS with 0.05% Tween-20 (Sigma)] for 5 min at room temperature. In situ reactions were performed with slight modifications as previously described (Weibrecht et al. 2013; Siwetz et al. 2016). For reverse transcription, 1 µM of each LNA primer (Exiqon) was added in the wells with 5 U/µl TranscriptMe Reverse Transcriptase (DNA-Gdansk; Gdansk, Poland), 1 U/µl RiboLock RNase inhibitor (Thermo Fisher Scientific), 0.5 mM dNTPs (Thermo Fisher Scientific), and 0.2 µg/µl BSA (NEB; Ipswich, MA, USA) to the room temperature Reaction Buffer (DNA-Gdansk). All units mentioned are displayed as final concentrations. Slides were incubated for 3 h at 45 °C in a humid chamber. After removing the reaction mix, the tissue was postfixed with 3% formaldehyde (Sigma) in DEPC-PBS for 10 min. After postfixation, slides were washed twice by flushing the Secure-seals chambers with PBS-T [DEPC-PBS with 0.05% Tween-20 (Sigma)] for 2 min each. The RNA-DNA hybrids formed by reverse transcription were degraded with ribonuclease H to make the target cDNA strands accessible for padlock probe hybridization. All reactions, ribonuclease H degradation, padlock probe hybridization, and ligation were performed in the same step. Ligation reaction was carried out with 0.5 U/µl Ampligase (Epicentre, Illumina, Madison, WI, USA), 0.4 U/µl RNase H (DNA-Gdansk), 0.2 µg/µl BSA (NEB), 0.1 µM of each padlock probe (Integrated DNA Technologies), and 50 mM KCl, 20% formamide (Sigma) in Ampligase buffer. The slides were incubated for 30 min at 37 °C and 45 min at 45 °C in a humid chamber. After the ligation step, the slides were washed by flushing the chambers once with 1× SSC-Tween and two times with 2× DEPC-PBS-T for 5 min each. The washing steps were followed by rolling-circle amplification (RCA) performed with 1 U/µl phi29 DNA polymerase (Thermo Fisher Scientific), 0.25 mM dNTPs (Thermo Fisher Scientific), 0.2 µg/µl BSA (NEB), and 5% glycerol (Sigma) in phi29 buffer. The incubation was carried out over-night at 37 °C in a humid chamber. The rolling-circle amplification incubation was followed by two washing steps with PBS-T [DEPC-PBS with 0.05% Tween-20 (Sigma)]. Rolling-circle products (RCPs) were visualized using 0.1 µM of each corresponding detection probe (Biomers) in 2× SSC and 20% formamide (Sigma) for 30 min at 37 °C in a humid chamber protected from light. Slides were washed two times with PBS-T [DEPC-PBS with 0.05% Tween-20 (Sigma)], nuclei were counterstained with 5 mg/mL DAPI (Thermo Fisher Scientific) for 5 min at RT. After DAPI staining, the reaction areas were marked at the bottom of the glass slide by scratching and secure-seals were removed from the slides. A series of 70, 85–99.5% (3 min each) ethanol was performed for dehydration. The samples were mounted with SlowFade Gold Antifade Mountant (Thermo Fisher Scientific) and stored at 4 °C protected from light until image analysis. A second tissue slide of normal colon tissue from the same patient was used for positive and negative controls. As an internal positive control, LNA primers and the padlock probe of the ubiquitously expressed β-actin (ACTB gene) transcript were added to the in situ reaction. LNA primers were not added to a second reaction spot as a negative control. Apart from these changes, all controls were treated with the same process as described above.

### Image acquisition and analysis

An HE stained section of the tumor tissue was used to ensure correct scoring of mutations in each tumor area, and the same four areas which were dissected for NGS sequencing were analyzed with the in situ detection method. As a control, non-neoplastic colon tissue from the same patient was used. Quantification was performed on ten 20× microscope images per area. As the tumor areas 1–4 have a different tumor cell content, images for quantification were taken within each area with a tumor cell content >80% to avoid bias by the surrounding stroma. Images were acquired using the Zeiss Observer.Z1 inverted microscope (Carl Zeiss; Oberkochen, Germany) equipped with a 120 W HXP Mercury short-arc lamp, a 2/3″ CCD camera (AxioCam MRm, Carl Zeiss) and an excitation and emission filter-set for visualization of DAPI, FITC, Cy3, and Cy5. A 20× objective (Plan-Apocromat, Zeiss) and the AxioVision software (Carl Zeiss, Version 4.8.2.0) were used for capturing the images. To ensure that all rolling-circle products (RCPs) were imaged, Z-Stacks were combined in one layer as a maximum intensity projection with the ZEN 2 blue software (Carl Zeiss, Version 2.0.0.0). The in situ overview image was generated by stitching of images using a 20× objective with the ZEN software. For better visualization, brightness and contrast of each image were adjusted. For visualization of specific RCP signals in the whole tissue overview, fluorescent signals which were visible in multiple wavelengths (unspecific signals) were subtracted from the respective detection channels. The FITC, CY3, and CY5 signals with a pixel intensity ratio of signal/background >5 were then enlarged by 50 or 100 pixels depending on the tissue size and number of RCP signals, and color coded to their respective transcripts using image editing software. For detailed quantification, signals with a pixel intensity ratio of signal/background >2 were manually inspected, counted, and false positive signals excluded [typically visible at multiple wavelengths (Weibrecht et al. 2013)].

### Statistical analysis

Statistical analyses were performed using the GraphPad Prism software, version 6.01 (GraphPad Prism, Inc., La Jolla, USA) for parametric comparison of two groups. An unpaired *T* Test was applied to compare the mean of the mutation allele frequency of every group (area 1 to area 4) to the mean of the non-neoplastic control tissue. Results were considered statistically significant when *p* < 0.05 (*p < 0.05, ***p < 0.001 and ****p < 0.0001).

## Results

### Detection of genetic heterogeneity in morphological homogeneous tumors by NGS

**Fig. 1:**
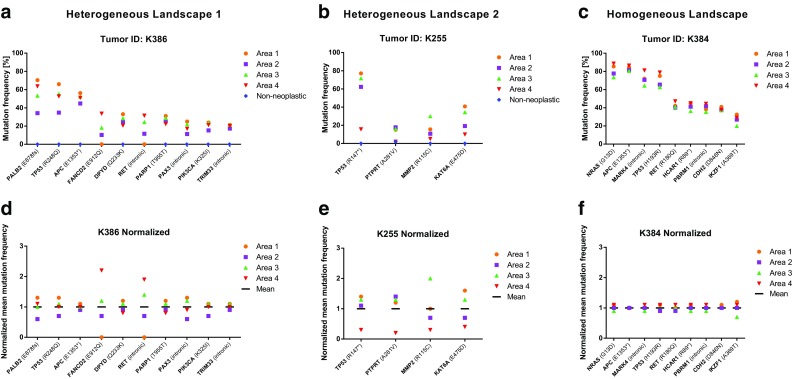
Genetic landscape of three tumors after microdissection into four separate areas. The *x-axis* represents the genes with detected mutations. The *Y-axis* reflects the mutation frequency of the corresponding genes in A, B, and C. **a** and **b** High degree of heterogeneity is indicated by different mutation frequencies in different tumor areas, such as FANCD2, RET, and KAT6A. **c** Tumor with a homogeneous genetic landscape evidenced by virtually identical mutation frequencies in each tumor area. Variability between different areas pertains solely to tumor cell content. **c, e**, and **d** For a better representation of genetic heterogeneity, the mean mutation frequency over all four tumor areas was calculated and set to 1. Each individual mutation frequency per area was normalized accordingly. Wide deviations from the mean mutation frequency, as seen in FANCD2, RET, or KAT6A, indicate genetic heterogeneity. Frameshift fs, stop codon = *

Mutational profiles of four separate areas of six colorectal carcinomas and their corresponding normal tissue were generated via next-generation sequencing. Comparison of the somatic mutation frequencies of several mutations over multiple tumor areas revealed in three out of six tumors (K229, K255 and K386) a high variability which was not explainable by simple differences in tumor cell content (Fig. 1, Supplementary Fig. 1). In the remaining three cases, the tumors represent genetically homogeneous tumors (Fig. 1, Supplementary Fig. 1). All tumors were assessed for microsatellite stability and found to be MSI stable.

The analysis of the sequence of 409 cancer-related genes in multiple areas (*n* = 4 per tumor) of morphological homogeneous tumors revealed a total of 34 somatic mutations in the cases investigated (Supplementary Table 2). 20 out of 34 (59%) were non-synonymous missense mutations, the remaining 14 mutations (41%) were either synonymous-, intronic-, or frameshift mutations. Further evaluation focused on samples with non-synonymous missense mutations which were differently distributed between the different tumor areas.

Two tumor samples with a differential mutational distribution between the tumor areas, which were not attributable to differences in tumor cell content, were re-evaluated by pyro-sequencing. In one of the two cases, the DPYD mutation c.697C > A was shown to be equally distributed in each tumor area by pyro-sequencing (Supplementary Fig. 2) arguing that technical differences in amplicon generation for NGS sequencing (i.e. differing amplification efficiencies of multiplexed PCR amplicon generation) might have been the cause for the observed mutant allele differences in the NGS analysis. However, in one case, NGS showed a different mutation frequency of the FANCD2 mutation c.2734G > C (Substitution–coding) over the four tumor areas investigated which was confirmed by pyro-sequencing (Supplementary Fig. 2).

### In situ mutation detection in colorectal tumor tissue

**Fig. 2:**
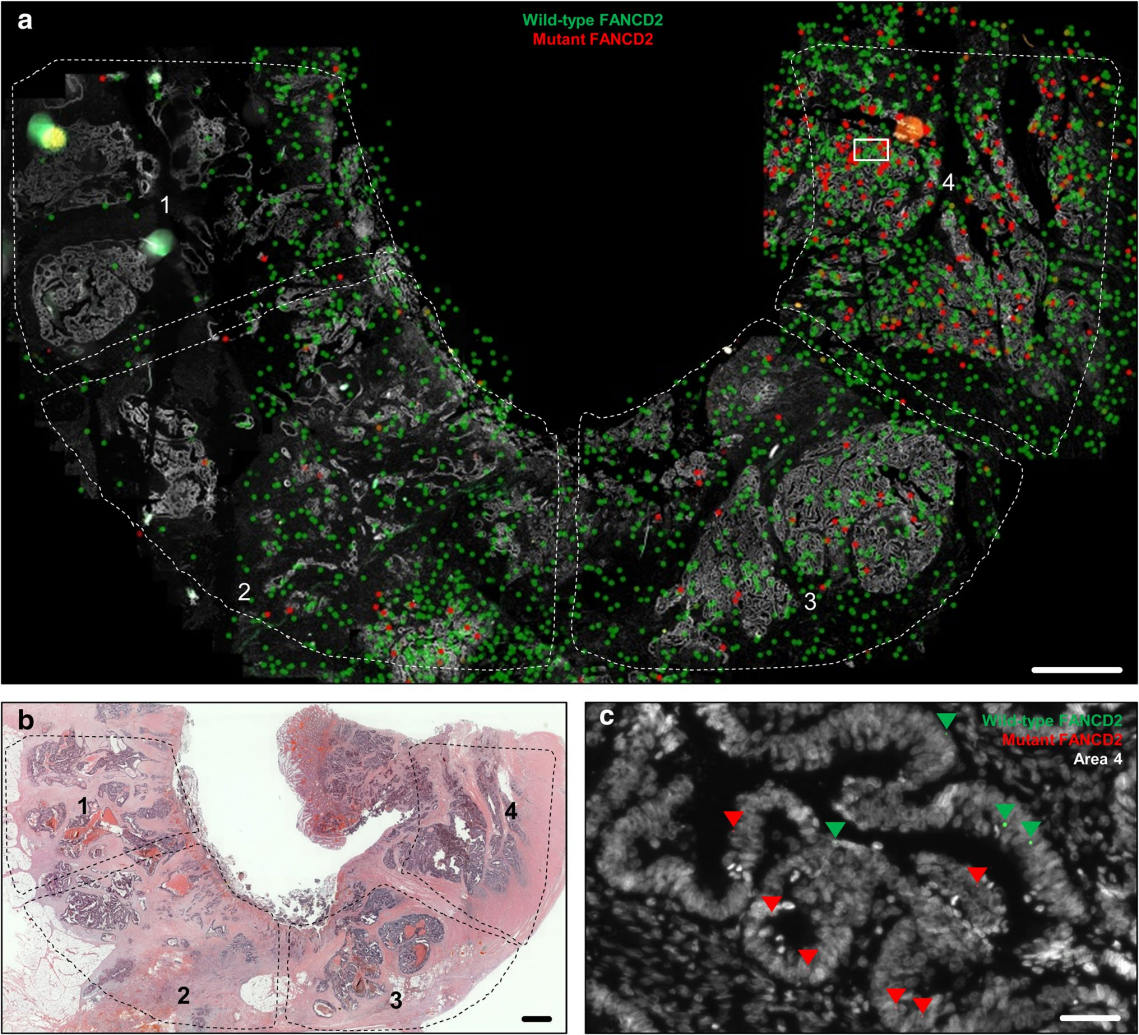
In situ mutation detection of FANCD2 wild-type and mutant transcripts in colorectal tumor tissue using padlock probes. **a** In situ location of transcripts in the four areas of interest. Each *dot* represents a transcript. *Green dots* are wild-type FANCD2; *red dots* are FANCD2 mutant transcripts. **b** Overview of the tissue in Hematoxylin and eosin staining. **c** Raw data image showing the locations of FANCD2 transcripts, magnified from the *boxed areas* in (**a**) area 4. *Arrowheads* depict wild-type FANCD2 (*green*) and mutant FANCD2 (*red*) transcripts. *Scale bars* in (**a**) and (**b**) represent 1 mm and in (**c**) 50 µm

**Fig. 3:**
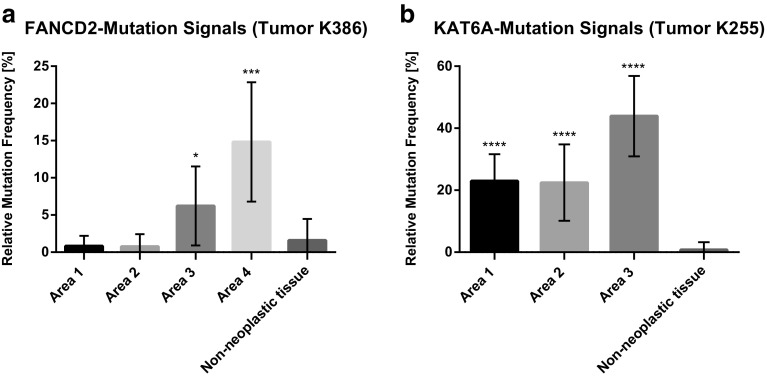
In situ mutation detection of FANCD2 and KAT6A mutations in a colorectal tumors. The relative mutation frequency indicates the ratio between wild-type and mutant signals in the respective tissue. **a** Quantification of transcripts in tumor K386 and **b** in tumor K255. Each data point represents the mean mutation frequency of ten 20× field of views of the respective tumor area with each field of view comprises >80% tumor content (*p < 0.05, ***p < 0.001, and ****p < 0.0001)

**Fig. 4:**
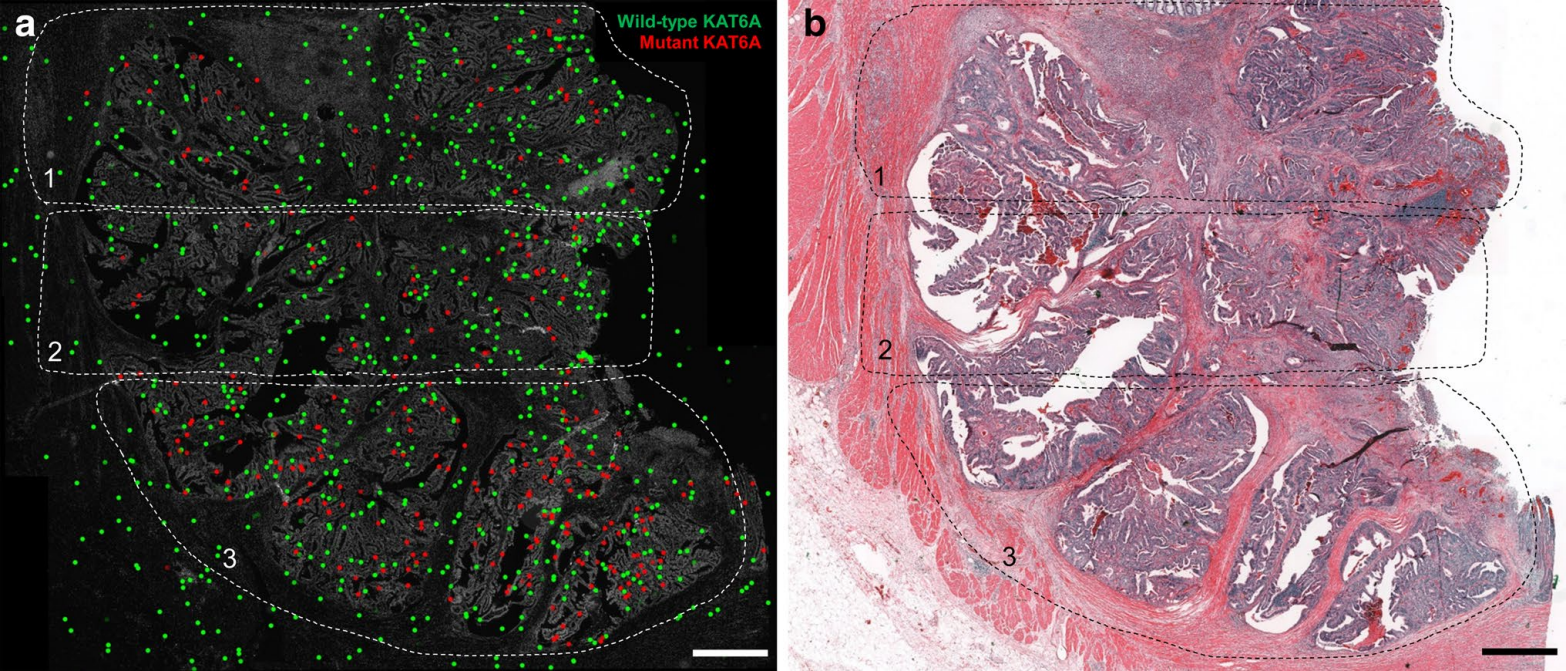
In situ mutation detection of KAT6A wild-type and mutant transcripts in colon tumor tissue using padlock probes. **a** In situ location of transcripts in the three areas of interest. **b** Overview of the tissue in HE. Each dot represents a transcript. *Green dots* are wild-type KAT6A transcripts, *red dots* are KAT6A mutant transcripts. *Scale bar* represents 1 mm

To localize the heterogeneous distribution of mutations in situ and to correlate its heterogeneous distribution with the corresponding HE topography of the tumor, we performed a mRNA-based in situ mutation detection approach based on padlock probes (Larsson et al. 2010). Padlock probes allow for visualization of single mRNA transcripts and can differentiate between two almost identical transcripts such as wild-type and mutant transcripts (Larsson et al. 2010). In situ mutation detection in tumor K386 showed an increased frequency of FANCD2 mutations from area 1 to area 4 (Figs. 2, 3a). In K386, all four tumor areas investigated displayed wild-type and mutant signals, whereas area 1 showed a lower amount of total in situ signals. Area 3 and Area 4 showed an increased mutant allele frequency compared to non-neoplastic colon control tissue (*p < 0.05, ***p < 0.001, respectively). The total number of mutant signals per area were 0.9% (3/342 signals; Mean 0.8%), 0.7% (2/295 signals; Mean 0.8%) 6.2% (17/273 signals; Mean 6.2%), and 13.4% (45/334 signals; Mean 14.8%) for area 1 to 4, respectively (Fig. 3a). Non-neoplastic colon tissue of the same patient showed a total number of FANCD2 mutant signal of 1.5% (4/262 signals; Mean 1.6%) suggesting a low unspecific technical background noise of the in situ methodology, in line with the previous published data (Larsson et al. 2010). In area 2, the in situ reaction revealed a second sub-clonal area of FANCD2 mutant signals. Quantification of the sub-area 2 resulted in a mutation frequency of 6.7% (25/371 signals). In tumor K225, the areas 1 till 3 were investigated by in situ mutation detection (Fig. 4). The area 4 was not included as it did not contain any tumor tissue in the deeper sections of the FFPE-block. The area 4 was depleted for NGS analysis. All three remaining areas had a significant higher mutant allele frequency compared to non-neoplastic colon tissue (****p < 0.0001) (Fig. 3b). The total number of mutant signals per area were 22.6% (57/252 signals; Mean 22.6%), 24.6% (50/203 signals; Mean 22.4), 45.0% (115/255 signals; Mean 42.7%) for area 1 to 3, respectively (Fig. 3b). Non-neoplastic colon tissue of the same patient showed a total number of 0.79% (1/127; Mean 0.90%) KAT6A mutant signals (Fig. 3).

## Discussion

The genetic landscape of six colorectal cancers with homogeneous morphology was evaluated by next-generation sequencing, pyro-sequencing, and a mRNA-based in situ approach. We investigated if spatially separate tumor areas harboured heterogeneous distributions of mutated cancer cells. Our data reveal that a morphological homogeneous tumor can have a heterogeneous genetic landscape. Parallel to sequencing technologies, in situ mutation detection is a powerful tool to visualize intratumor heterogeneity within the architecture of a tumor.

### NGS shows heterogeneity but needs confirmation by a second sequencing technology

NGS allows for the sequence analysis of many genes concurrently. This leads to a much higher mutation detection rate than the traditional sequence analysis methods. In our study, up to 10 mutations could be identified in a single tumor using our comprehensive cancer panel, which is in accordance to our in house routine sequencing panel of colorectal cancers (data not shown). In comparison, “The Cancer Genome Atlas” (TCGA) study of colon and rectal cancers identified a mean number of 58 somatic mutations per tumor, but based on exome sequencing of non-hypermutated tumors (Muzny et al. 2012). The allele frequencies of the detected mutations can, however, vary depending on multiple factors such as differences in PCR (or hybridization) efficiency, sequencing artifacts, and also genetic heterogeneity. Our data reveal that apparent genetic heterogeneity of tumor areas may also arise due to technical artifacts. Re-examination of mutation frequencies with an independent quantitative assay (pyro-sequencing) revealed technical artifacts as the reason for the heterogeneity detected by NGS in one case, but confirmed genetic heterogeneity in another. To reliably determine genetic heterogeneity, it is thus important to confirm NGS results with an independent method, at least for amplicon-based NGS technologies.

### In situ mutation detection reveals heterogeneity

Our results are largely in agreement with a recent study by Hardiman et al. (2016). They investigated six rectal carcinomas using whole-exome sequencing followed by deep targeted sequencing with Ion Torrent and Oncoscan SNP arrays to assess copy number alterations and found substantial genetic heterogeneity (Hardiman et al. 2016). In our approach, we included colon as well as rectal cancers and performed an in situ approach directly on the tumor tissue. The mRNA-based in situ approach by padlock probe technology allows direct visualisation of mutated transcripts on tissue sections, and is referred to as in situ mutation detection (Grundberg et al. 2013). The key strength of in situ mutation detection is the ability to localize mRNA transcripts directly in the tissue retaining the spatial information. Furthermore, padlock probes are highly specific, allowing to differentiate sequences which differ by only a single base (e.g.: mutant vs wild type) (Larsson et al. 2010). In contrast, by applying next-generation sequencing, tissues are lysed to extract nucleic acids. Therefore, the histological context of the tissue is lost. Moreover, the nucleic extracts represent several thousand cells and we have no understanding of where the different sub-clones reside within the tissue in a geographical sense. In situ mutation detection can close the gap between sequencing data and spatial information.

By in situ mutation detection, we could clearly localize FANCD2 wild type as well as mutated FANCD2 transcripts to certain tumor areas with an increased mutation allele frequency from area 1 to area 4. Remarkably, in situ mutation detection could clearly identify a sub-area in area 2 which contains the FANCD2 mutated cells (Fig. 2a). The morphological appearance of the sub-area in area 2 did not differ from that of other epithelial neoplastic areas of the respective tumor. Also KAT6A mutation transcripts showed a higher number of mutant signals in area 3, indicating genetic heterogeneity. In general, wild-type transcripts were detectable in the epithelial as well as the stromal compartments, whereas the mutant transcripts were exclusively localized to neoplastic epithelial tissue (apart from minor technically inherent background signals). This finding can be explained by the native in vivo protein function of the investigated targets KAT6A and FANCD2. The FANCD2 protein is involved in the pathway for DNA inter-strand crosslinks repair (Kozekov et al. 2003), and is, therefore, not just expressed in the epithelium, but also in the stromal compartments of a tissue. The malfunction of this repair mechanism leads to accumulation of mutations and is associated with the early cancer development and genomic instability (Reliene et al. 2010; Shen et al. 2015; Michl et al. 2016). In general, genomic instability is considered to be the main driving force for genetic tumor heterogeneity. Genomic instability can be caused by many functional deficiencies, such as dysfunction of nucleotide repair mechanisms, poor telomere maintenance, deregulated DNA replication, as well as defects in chromosomal segregation (Burrell et al. 2013). KAT6A protein belongs to the family of histone acetyltransferases and is expressed in all tissues (Uhlen et al. 2015) (Human Protein Atlas available from http://www.proteinatlas.org). KAT6A mutations were widely distributed with the highest mutant allele frequency in area 3. The spatially distribution of heterogeneous mutation has mostly been envisioned as contiguous. These contiguous, sub-clonal mutation areas would be owned to the expansions of sub-clonal mutations sweeping and replacing adjacent clones by virtue of their dominant proliferation. Recently Sottoriva et al. have questioned that assumption by providing evidence through mathematical modelling and confirmatory molecular analysis from actual tumor tissue, that sub-clonal heterogeneity in colorectal cancer is rather dispersed in a “big bang” like fashion across the tumor (Sottoriva et al. 2015). This expansive distribution would lead to sub-clonal areas corresponding in size to the age of the clone and—equally important—can be randomly distributed over different, spatially separate, and remote areas of invasive cancers with irregular topographic outlines of the sub-clones. By microdissection and subsequent molecular analysis, the authors were able to map out sub-clonal distributions strikingly reminiscent to the distributions visualized by in situ mutation detection in our study, especially in case of KAT6A mutations (Fig. 4), where the sub-clonal mutation shows multiple interspersed areas within (“folded into”) surrounding areas of KAT6A wild-type neoplastic epithelium. In case of FANCD2, the mutation bearing clones are predominantly located in comparatively small tumor areas (area 3, area 4, and focally in area 2). According to the big bang model, the FANCD2 mutation is a late mutation in this tumor, as otherwise, it would be equally distributed over all tumor areas.

## Final conclusion

Genetic heterogeneity of morphologically homogeneous colorectal cancer seems to be a frequent event. Unusual frequencies of mutated alleles not in keeping with the estimated tumor cell content of the areas sampled, can be attributed to sub-clonal genetic heterogeneity in a subset of cases. Although we analyzed only a limited number of samples, our study demonstrated that morphological tumor homogeneity does by no means exclude genetic tumor heterogeneity. It is safe to assume that genomic tumor heterogeneity is an underreported phenomenon, considering inherent methodological detection limits and analysis from only parts of the whole tumor in most cases. Sensitive and specific detection of tumor heterogeneity depend on careful morphological and genetic correlations and an appropriate selection of methodologies. To that end, in situ mutation analysis-in our opinion-readily deserves a place in this arsenal of options as it is inherently well suited to the task of bridging morphology with genomics.

## Electronic supplementary material

Below is the link to the electronic supplementary material.

Genetic landscape of three tumors determined by NGS. The x-axis represents the genes with detected mutations. For a better representation of genetic heterogeneity, the mean mutation frequency over all four tumor areas was calculated and set to 1. Each individual mutation frequency per area was normalized accordingly. Wide deviations from the mean mutation frequency indicate genetic heterogeneity. A) represents a genetically heterogeneous and B) and C) genetically homogeneous tumors. The mutation in the gene DPYD (R886H) of the tumor K229 in A) was re-evaluated by pyro-sequencing and yielded a homogeneous distribution in contrast to the NGS data (see Supplementary Fig. 3). Framshift = fs, stop codon = * (EPS 29 KB)

Re-evaluation of next-generation sequencing (NGS) data by pyro-sequencing. NGS identified DPYD (c.G2657A) and FANCD2 (c.G2734C) to be differentially abundant in the four different tumor areas of two different tumors. For DPYD, pyro-sequencing revealed no differences in mutation frequencies between tumor areas 1 till 4. The FANCD2 mutation was confirmed by pyro-sequencing to be increasingly abundant ranging from tumor area 1 till 4 (EPS 47 KB)

Supplementary material 3 (XLSX 11 KB)

Supplementary material 4 (XLSX 17 KB)
